# Solvent Evaporation-Induced Self-Assembly of Flexible Cholesteric Liquid Crystal Elastomers: Fabrication, Performance Tuning, and Optimization

**DOI:** 10.3390/ma18091927

**Published:** 2025-04-24

**Authors:** Jinying Zhang, Yexiaotong Zhang, Zhongwei Gao, Jiaxing Yang, Xinye Wang

**Affiliations:** 1 Beijing Key Lab for Precision Optoelectronic Measurement Instrument and Technology, School of Optics and Photonics, Beijing Institute of Technology, Beijing 100081, China; 3120220661@bit.edu.cn (Y.Z.); 3220230637@bit.edu.cn (Z.G.); 3120220654@bit.edu.cn (J.Y.); 3120205353@bit.edu.cn (X.W.); 2 Yangtze Delta Region Academy, Beijing Institute of Technology, Jiaxing 314001, China; 3 National Key Laboratory on Near-Surface Detection, Beijing 100081, China

**Keywords:** liquid crystal elastomers, self-assembly, structural color

## Abstract

The realization of broad-wavelength tunability of the structural color in Double layered Cholesteric Liquid Crystal Elastomers (DCLCEs), along with good flexibility and processability, presents a significant challenge. This research introduces a facile and effective fabrication technique, Solvent Evaporation-Induced Self-Assembly (SEISA), for the production of DCLCEs exhibiting broad wavelength tunability, superior flexibility, and robust mechanical characteristics. Focusing on initial color tuning, bubble defect minimization, UV photopolymerization, and coating procedures, this research systematically optimizes the fabrication process through experimental investigation of factors like chiral dopant amount, temperature, UV exposure duration, coating thickness, and speed. The method enabled the successful fabrication of DCLCEs with uniform and controllable coloration, demonstrating the effectiveness of this controlled synthesis approach in significantly enhancing structural color features. Upon stretching to 2.8 times its original length, the center wavelength shifted from 613 nm to 404 nm, yielding a tunable bandwidth of up to 209 nm across the visible spectrum.

## 1. Introduction

Nature’s abundant and intricate display of colors in organisms offers a continuous stream of inspiration for the design and development of bioinspired structural color materials. Conventional methods for realizing color rely primarily on chemical dyes or pigments; however, these materials are often associated with issues such as fading, toxicity, and limited controllability. Unlike chemical coloration, structural color materials generate vibrant and diverse colors through the precise control of nanostructure interactions with light. Structural color materials exhibit numerous advantages such as color vibrancy, durability, non-toxicity, and tunability, showing significant potential for application across various fields [1,2,3,4]. Drawing inspiration from the structural colors found in various organisms (e.g., butterfly wings [5], peacock feathers [6], chameleon skin [7,8]), researchers have explored a wide range of structural color materials, including photonic crystals [9,10], multilayer films [11,12], and cholesteric liquid crystals [13,14,15]. These structural color materials have shown great application potential in fields such as optical displays [16], sensors [17], anti-counterfeiting technology [18], smart coatings [19], and biomedicine [20].

Cholesteric liquid crystals (CLCs), owing to their unique helical molecular arrangement, selectively reflect light of specific wavelengths, thereby exhibiting vibrant structural colors. Cholesteric liquid crystal elastomers (CLCEs) materials not only preserve the optical properties of CLCs but also endow the material with excellent mechanical properties and tunability. The fabrication and performance optimization of CLCEs still face numerous challenges. Conventional fabrication methods for CLCEs typically necessitate complex processes or equipment, hindering their large-scale application [21]. Furthermore, achieving broadband wavelength tunability of the structural color of CLCEs while imparting good flexibility and processability remains a challenging issue. The color variation in CLCEs is generally constrained by both the initial helical pitch and the film’s mechanical properties. Upon stretching, the film thickness decreases, and the helical pitch (p) is compressed, resulting in a blueshift of the reflected wavelength. However, due to the limited range of change in the helical pitch, the optical response of the film largely depends on its initial reflection wavelength (initial color). Furthermore, the formation of bubble defects during the fabrication of CLCEs is another critical issue that significantly affects their optical performance and mechanical stability. Even at the micron scale, these bubbles significantly scatter incident light, diminishing the film’s reflectivity and color purity, and potentially causing stress concentration, thus impacting the film’s durability. In addition to optical properties, the mechanical properties of CLCEs are also key factors determining their application feasibility. In practical applications, CLCEs need to withstand various external forces, and their degree of polymerization and crosslinking density also affect the strength, toughness, and elasticity of the film. Furthermore, the thickness uniformity of CLCEs also has a significant impact on their optical and mechanical properties; uneven films can lead to uneven color, stress concentration, and other issues. To address these issues, researchers are continually exploring new CLCEs fabrication methods and material systems. For instance, by modifying crosslinking agents and chain extenders, the network structure and helical pitch of CLCEs can be tuned, thus enabling control over their optical properties [22,23]. However, most studies still focus on the fabrication of single-layer CLCEs on rigid substrates; how to effectively combine CLCEs with flexible substrates and maintain its excellent optical and mechanical properties still requires further research.

To address the above-mentioned challenges, this study introduces a facile and efficient fabrication method, Solvent Evaporation-Induced Self-Assembly (SEISA), for the preparation of double layered CLCEs (DCLCEs) exhibiting broadband-wavelength tunability, excellent flexibility, and robust mechanical properties. The method optimizes the color change range of the DCLCEs through precise control of their initial color. By precisely controlling the concentration of the chiral dopant LC756, we successfully achieved control over the initial helical pitch of the DCLCEs, thereby achieving controllable adjustment of the initial reflection color from red to blue. Additionally, this research delves into the formation mechanism of bubble defects and proposes effective strategies for their control. Bubble formation was suppressed by systematically studying the influence of coating temperature and optimizing process parameters. Concurrently, we systematically studied the effect of ultraviolet (UV) irradiation dosage on the mechanical properties of DCLCEs, elucidating the relationship between UV irradiation dosage and Young’s modulus, tensile strength, and elongation at break. To obtain DCLCEs with uniform thickness, we employed the blade coating method and systematically optimized the blade coating process parameters (blade height and coating speed). This study employed a novel DCLCEs precursor system based on a two-step Michael addition reaction. By coating this precursor solution onto a flexible elastomer substrate and utilizing the solvent evaporation-induced self-assembly process, DCLCEs with a mono-domain nano-helical structure were successfully fabricated. This method does not require complex equipment or processes and is simple to operate. The use of double-sided tape as a flexible substrate, with its inherent microstructure, facilitated the formation of a uniform monodomain structure. The fabricated DCLCEs exhibited pronounced color changes upon mechanical stretching, with the reflection wavelength undergoing a blueshift from 613 nm to 404 nm, demonstrating a broad tunable bandwidth. This study provides a new approach for the development of novel flexible displays, sensors, and smart color-changing films.

## 2. Experiment

### 2.1. Preparation of CLCE Precursors

**Figure 1:**
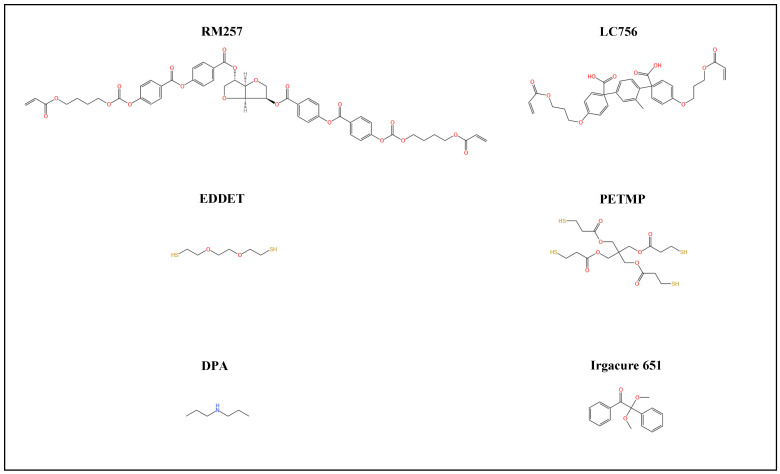
Materials for the synthesis of the CLCE precursor.

First, 4.1 wt.% of the chiral reactive mesogen dopant 2,5-Bis-O-[4-[[4-[[[4-(acryloyloxy)butoxy]carbonyl]oxy]benzoyl]oxy]benzoyl]-1,4:3,6-dianhydro-D-glucitol (LC756, Macklin, Shanghai, China) was premixed in the achiral diacrylate reactive mesogen 1,4-Bis-[4-(3-acryloyloxypropyloxy)benzoyloxy]-2-methylbenzene (RM257, Macklin, Shanghai, China). The prepared chiral premix was dissolved in toluene (Macklin, Shanghai, China) at a concentration of 50 wt.% and 80 °C for 10 min with magnetic stirring at 500 rpm. The thiol chain extenders (dithiol monomer 2,2′-(ethylenedioxy)diethanethiol) (EDDET, Macklin, Shanghai, China) and pentaerythritol tetrakis(3-mercaptopropionate) (PETMP, Aladdin, Shanghai, China), and photoinitiator (2,2-dimethoxy-2-phenylacetophenone) (Irgacure 651, Aladdin, Shanghai, China) were added to the prepared chiral premix solution. EDDET possesses two thiol groups (-SH) that can undergo efficient Michael addition reactions with acrylate groups (-C=C-COOR), achieving crosslinking of the liquid crystal molecules. PETMP is a multifunctional thiol monomer that can undergo Michael addition reactions with the acrylate groups mentioned in the previous steps, further enhancing the crosslinking density and mechanical properties of the material. Next, a diluted catalyst solution consisting of dipropylamine (DPA, Macklin, Shanghai, China) and toluene was added to the solution at a weight ratio of 1:50 and vigorously stirred for 5 min to obtain the final CLCE precursor. The precursor solution was degassed in a vacuum oven at 25 °C (room temperature) under a vacuum of 508 mmHg for 10 min to eliminate any bubbles formed during mixing. Table 1 shows the amounts of each precursor component used for each batch of CLCEs. Figure 1 shows the materials of the finished CLCE precursor.

In this study, 3M™ VHB™ 4910 double-sided tape was used as the flexible substrate. This tape is a 1.0 cm thick acrylic foam double-sided tape with a polyethylene (PE) release liner. It was chosen as the flexible substrate due to its good flexibility and the mechanical properties of a hyperelastic material. The tape was cut to dimensions of 5 cm × 10 cm, and the surface was cleaned with anhydrous ethanol and allowed to air-dry at room temperature for 10 min. Afterward, the trimmed substrate was affixed to a level experimental platform. During the self-assembly process of the DCLCEs chiral precursor solution, the microstructure of the flexible substrate restricted the evaporation direction of the toluene solvent, thereby exerting a strong anchoring effect on the self-assembly of the helical nanostructures. Notably, the double-sided tape served as the bottommost supporting layer in the bilayer film structure within the context of this study.

**Figure 2:**
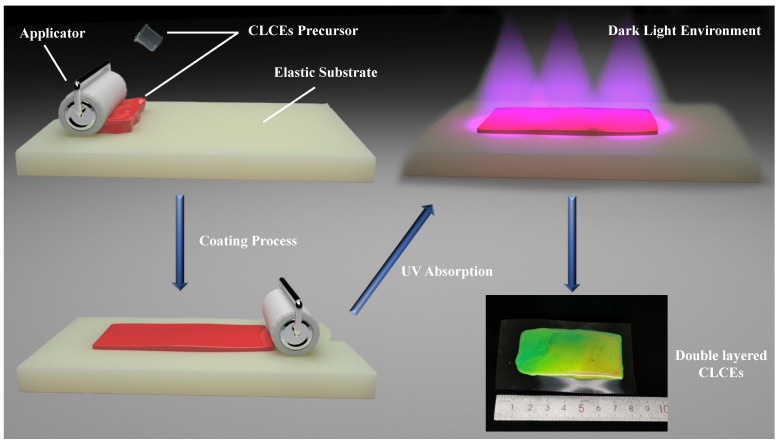
Schematic of the precursor coating process. The CLCE precursor is dispensed onto the elastic substrate, and a blade performs uniform coating at a constant speed and height, following a resting period for solvent evaporation, photocuring is accomplished through ultraviolet light exposure. The blue arrows in the figure represent the sequence of the coating process.

Coating is a widely used thin film preparation technique that involves applying a viscous liquid material onto the surface of a substrate and allowing it to spread evenly, producing a thin film with specific thickness and properties. Blade coating utilizes a movable blade to evenly spread the precursor layer to the target thickness. During the blade coating process, the movement of the blade applies significant shear force to the CLC material, which is crucial for promoting the self-assembly of the cholesteric liquid crystal. The shear force induces the material’s molecules to tend towards an ordered arrangement along the direction of the substrate surface within a short period, and promotes the formation of the cholesteric helical structure. Figure 2 illustrates the curing process of the precursor into a double-layered CLCEs via the coating method. The precursor, after degassing, is positioned between the flexible substrate and the coating blade for unidirectional coating. After the coating is completed, the precursor needs to stand for 24 h to allow the solvent to evaporate, and then be cured by ultraviolet light irradiation. In the photocuring process, the photoinitiator absorbs UV light, generating free radicals that initiate polymerization and crosslinking reactions of monomers and oligomers, ultimately resulting in a stable, double-layered CLCEs.

**Figure 3:**
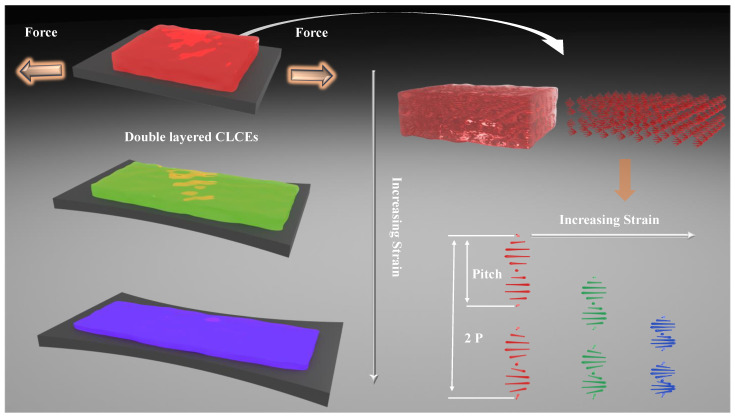
Selective reflection induced by macroscopic and microscopic structural alterations upon stretching of DCLCEs. As the DCLCEs are stretched, their thickness decreases and the helical pitch shortens, causing the reflective structural color to blue-shift from red to green to blue.

Cholesteric liquid crystal molecules are arranged in a unique helical structure, with regular layered arrangements. Within each layer, the liquid crystal molecules are parallel to each other, with their long axes parallel to the plane of the layer. Between adjacent layers, there is a certain angle between the liquid crystal molecules, causing the direction of the long axis of the molecules to change helically along the normal direction of the layers. CLCs can be considered a specific type of nematic liquid crystal. As the angle between liquid crystal molecules in neighboring layers progressively changes from 0° to 360° along the helical structure direction, the molecular arrangement returns to its original state, completing a full helical period. This periodic interlayer distance is called the pitch (p) of the CLCs, and its size is usually between tens of nanometers and several micrometers, as shown in Figure 3. The selective reflection of CLCs originates from their unique molecular helical arrangement. Selective reflection occurs when the incident light wavelength and the helical structure fulfill the Bragg reflection condition, expressed as λ=n×p (where λ represents the central wavelength of the reflection spectrum, n is the average refractive index of the liquid crystal, and p is the pitch). Macroscopically, in the liquid crystal elastomer, stretching the liquid crystal causes its length to increase, its thickness to reduce, the pitch to shorten, and consequently, the reflected light shifts towards shorter wavelengths. Conversely, when the stress is unloaded, causing it to contract, the pitch increases, and the structural color of its selective reflection moves towards the long-wavelength direction until it returns to the initial color. This process can be described by the following equation:

(1) $$\Delta \lambda =n\times \Delta p=n(p_{0}-p_{1})=n(p_{0}-p_{0}\frac{d_{1}}{d_{0}})$$

where Δλ represents the shift in the central wavelength of the CLCE reflection spectrum, $p_{0}$ denotes the initial pitch of the unstretched CLCE, $p_{1}$ is the pitch after stretching, $d_{0}$ signifies the initial thickness, and $d_{1}$ is the altered thickness. This equation establishes the connection between the selective reflection wavelength shift and the elastomer’s thickness variation. This unique functionality makes them well-suited for a wide range of applications requiring versatility and responsiveness.

### 2.2. Initial Color of DCLCEs: Precise Control of Nano Helical Structures

The color-changing range of DCLCEs is influenced by both the initial pitch and the mechanical properties; optimizing this range is of paramount importance for enhancing their value in practical applications. Stretching DCLCEs results in a reduction of their thickness and compression of the pitch, thereby inducing a blueshift in the reflected wavelength. Due to the limited range of thickness variation, the optical response of the film is generally constrained by its initial reflection wavelength (initial color). For example, when the initial reflection wavelength of the DCLCEs is in the red region, the color-changing range covers the red, green, and blue regions during the stretching process. Conversely, if the initial reflection wavelength is located in a shorter wavelength region (such as blue), the color-changing range of the film during stretching may be severely compressed, limiting the breadth of its optical dynamic change. Therefore, precisely tuning the initial color of the DCLCEs, so that its pitch and reflection wavelength are within an appropriate starting range, is the key to achieving a wide range of color changes and optimizing the mechano-optical properties. Furthermore, the application scenario and specific function of the DCLCEs also determine the initial color. A wide range of color tuning from red to blue light is suitable for fields such as dynamic camouflage, flexible displays, and structural color-changing pigments; While for applications requiring small-range color changes and high sensitivity, such as micro-deformation detection, an excessively wide change range will reduce the response speed of the device. Therefore, the targeted design of the initial reflection wavelength is of great significance for different application requirements.

In CLCs, the initial pitch of the helical structure is significantly affected by the ratio of the liquid crystal host and the chiral dopant. The chiral induction effect of the chiral dopant exerts a controlling effect on the alignment angle and twisting strength of the liquid crystal molecules, and its concentration directly affects the pitch size, which further determines the central wavelength of the reflected light. The Helical Twisting Power (HTP) is a parameter that quantitatively describes the ability of a chiral agent to induce twisting of liquid crystal molecules. The HTP value is defined as:

(2) $$p=\frac{1}{c\times HTP}$$

where p is the pitch and c is the concentration of the chiral dopant. The concentration of the chiral dopant is a key factor affecting the pitch. Within a certain concentration range, the pitch is usually inversely proportional to the concentration of the chiral dopant. In this study, RM257 serves as the host material, providing the basic structure and matrix of the liquid crystal, and its molecular structure contains rod-like liquid crystal mesogens. When the concentration of RM257 is sufficiently high, these liquid crystal mesogens spontaneously align to form a liquid crystal phase, providing the basis for the formation of the cholesteric phase. LC756 is a chiral molecule that interferes with the arrangement of RM257 liquid crystal mesogens, forming a helical structure, thus forming the cholesteric phase. The amount of LC756 added affects the pitch of the cholesteric phase, that is, the periodic length of the helical structure. The magnitude of the pitch determines the wavelength of light selectively reflected by the cholesteric liquid crystal, and thus determines its color.

**Figure 4:**
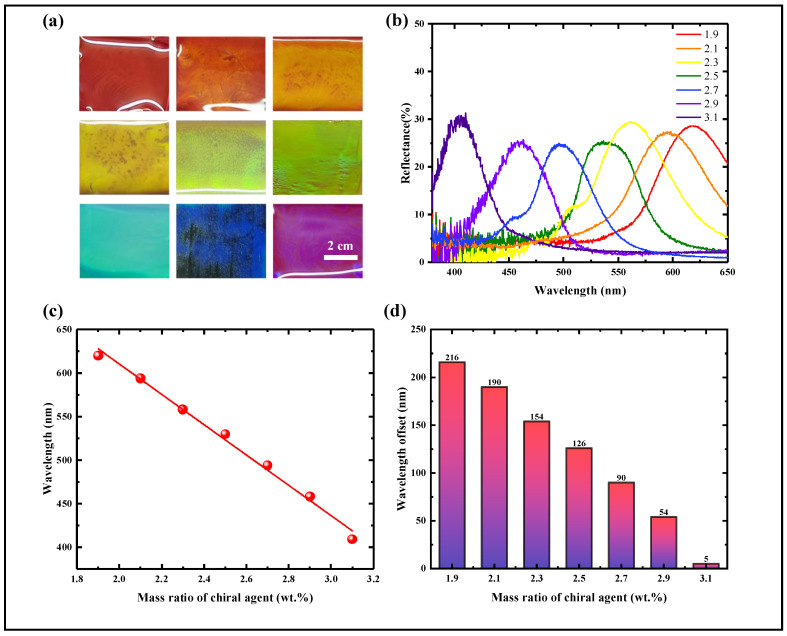
Initial color control via chiral agent concentration modulation. (**a**) CLCE samples with different initial colors. (**b**) Spectral curves showing the addition of LC756 from 1.9 wt.% to 3.1 wt.%. (**c**) The trend of change in the central wavelength of the reflection spectrum as the amount of LC756 added is from 1.9 wt.% to 3.1 wt.%. (**d**) The range of central wavelengths of the reflection spectrum covered when stretched, with the addition of LC756 from 1.9 wt.% to 3.1 wt.%.

By adjusting the mass fraction of the chiral dopant LC756, controllable tuning of the initial reflection color of DCLCEs from red to blue was achieved (Figure 4a). By adjusting the mass ratio of the liquid crystal host RM257 and the chiral dopant LC756, we systematically investigated the effect of the dopant concentration on the initial reflection wavelength of DCLCEs. The experimental results show that with the increase of dopant concentration (c), the helical pitch (p) of CLCE shows a significant shortening trend, which in turn leads to a shift of the central wavelength of reflected light towards the short-wave direction (Figure 4b,c). For CLCE with a red reflection color, the mass fraction of LC756 is 1.9 wt.%; when the mass fraction is increased to 2.5 wt.%, the initial reflection color of CLCE changes to green; And when the mass fraction of LC756 is further increased to 2.9 wt.%, the initial reflection color of CLCE becomes blue. These results indicate that the concentration of the chiral dopant LC756 directly determines the helical pitch length of the DCLCEs, thereby controlling its initial reflection wavelength and color.

Uniaxial tensile tests were performed on DCLCEs with different initial colors to systematically study their dynamic color change range and behavior under external force. DCLCEs with different initial reflection wavelengths all exhibit a blue shift of the reflection peak wavelength during the stretching process, but their color change range and the covered spectral region are significantly affected by the initial color. The initial red light sample (λ≈ 613 nm) exhibited a blue shift in the reflection wavelength from 613 nm to 404 nm in the stretching strain range of 0% to 180%, covering a spectral range of 209 nm (613 nm–404 nm). The spectral ranges covered by the initially green sample (λ ≈ 535 nm) and the initially blue sample (λ ≈ 460 nm) were 121 nm and 46 nm, respectively. The results indicate that a longer initial wavelength corresponds to a broader color change range after stretching, while a shorter initial wavelength results in a narrower color change range (Figure 4d). This observed trend demonstrates that the film’s initial reflection wavelength is a crucial factor determining its overall dynamic tunability. 

### 2.3. Bubble Defects in DCLCEs: An Analysis of Formation Mechanisms and Control Strategies

**Figure 5:**
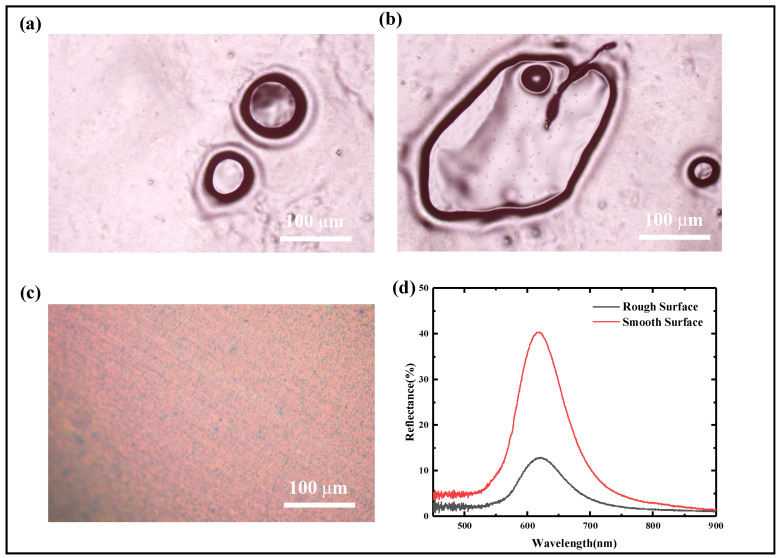
Bubble defects in DCLCEs. (**a**,**b**) The morphology of bubbles on the DCLCEs under the microscope is mostly circular or elliptical, with a small number of irregularly shaped distributions. (**c**) Smooth surface of the DCLCEs after process improvement. (**d**) Spectral curves of smooth and rough surfaces (with and without bubbles).

The production of high-quality DCLCEs is essential for their applications in fields such as optics and mechanics. However, the formation of bubbles during the fabrication of DCLCEs is a persistent challenge. These bubble defects, even at the micrometer scale, can significantly scatter incident light, reducing the optical performance of the film, and can potentially lead to stress concentrations, impacting its mechanical stability. To fully exploit the excellent properties of DCLCEs, it is essential to minimize or eliminate bubble defects. This section will provide a detailed analysis of the causes of bubble formation during DCLCEs preparation and propose effective control strategies, with the goal of fabricating bubble-free, high-performance DCLCEs. To investigate the influence of bubble defects on DCLCEs, we first conducted a detailed characterization of unoptimized DCLCE samples. As shown in Figure 5a–c, observations using an optical microscope (ECLIPSE LV100D, Nikon Corporation, Tokyo, Japan [100× & 200×]) revealed the presence of numerous bubbles on the film surface; these bubbles were mostly circular or elliptical in shape, with a wide diameter distribution ranging from 20 μm to 10,000 μm.

To quantitatively evaluate the influence of bubbles on the optical properties of the film, we measured the reflection spectra of DCLCEs with and without bubbles (or with few bubbles) using a spectrometer (PG4000, Ideaoptics, Shanghai, China). As shown in Figure 5d, the bubble-free DCLCEs exhibited a clear, sharp reflection peak with a maximum at 620 nm, demonstrating good selective reflection characteristics. In contrast, the reflection peak intensity of the DCLCEs containing a large number of bubbles was significantly reduced. The results indicate that the presence of bubbles not only reduces the reflectivity of the film but also disrupts the uniformity of the CLC helical structure, leading to a decrease in selective reflection capability and a reduction in color purity.

The bubbles affecting the smoothness of the DCLCEs mainly originate from the evaporation process of the solvent (toluene) after coating. Excessively fast or slow solvent evaporation can both lead to defect formation, affecting the uniformity of the structural color. During this process, controlling the temperature and the amount of solvent used have a significant influence on bubble formation. To investigate the influence of temperature on bubble formation during the coating process of DCLCEs, we conducted a series of controlled experiments. Experiments were conducted on a hot plate (WY-01B, Labshark, Beijing, China) equipped with precise temperature control, simulating various coating temperature environments. The DCLCE precursor solution was prepared as previously described, using toluene as the solvent. Clean elastic substrates were placed on the hot plate. The hot plate was set to the target temperatures: 20 °C, 30 °C, 40 °C, 50 °C, and 60 °C, and preheated for 30 min to allow the substrate and the hot plate to reach thermal equilibrium. The DCLCE precursor solution was coated onto the substrate using a coating method. After coating, the samples were kept on the hot plate, maintaining the set temperature, until the solvent (toluene) had completely evaporated, and the DCLCEs was cured by UV irradiation. Image analysis of the microscope photographs was performed using ImageJ software (Version 1.8.0) to quantitatively assess the bubble density (number of bubbles per unit area), bubble area fraction (ratio of total bubble area to total film area), average diameter, and size distribution.

**Figure 6:**
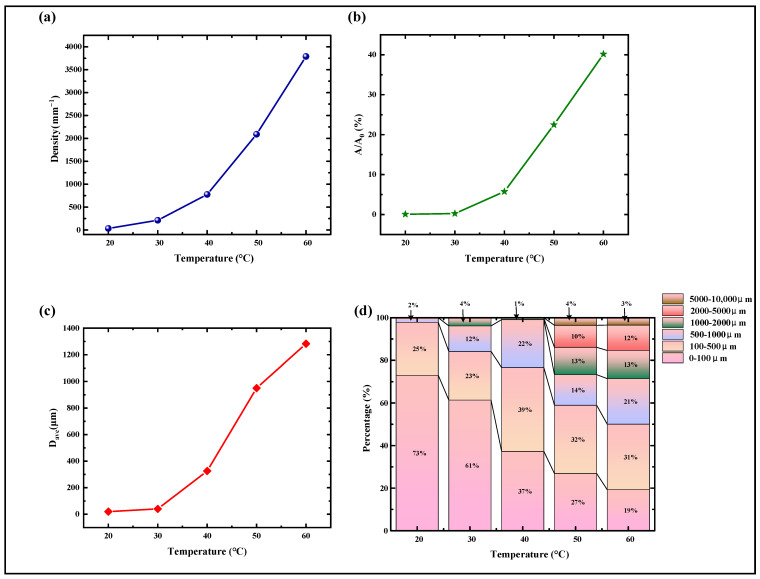
Quantitative characterization of bubbles at different temperatures. (**a**) Bubble density (number of bubbles per unit area). (**b**) Bubble area fraction (ratio of total bubble area to total film area). (**c**) Average diameter. (**d**) Size distribution.

We calculated the bubble density, which is the number of bubbles per unit area. By using image analysis software (ImageJ, Version 1.8.0), edge detection and segmentation were performed on the grayscale images to count the number of bubbles in each region. The formula for calculating the average bubble density is the total number of bubbles divided by the total area. As shown in Figure 6a, the bubble density generally showed an increasing trend with increasing experimental ambient temperature, especially above 30 °C, where the upward trend became more pronounced, and the bubble density increased significantly.

Since bubble density does not reflect the size and distribution of bubbles, we also calculated the bubble area ratio, which is the percentage of the total bubble area to the total film area, in order to more comprehensively characterize bubble defects. Using image analysis software, the microscope photographs were binarized to identify the bubble regions, and the total area of the bubble regions was calculated. The formula for calculating the bubble area ratio is: (total bubble area/total film area) × 100%. As shown in Figure 6b, the bubble area ratio generally showed an increasing trend with increasing experimental ambient temperature, with a significant increase in the area covered by bubbles, and the change was obvious.

We also calculated the average bubble diameter and size distribution (Bubble Size Distribution), which describes the distribution of the number of bubbles of different sizes. The diameter (or equivalent diameter) of each bubble was measured using image analysis software. It was statistically found that the bubbles in the DCLCEs mainly originate from the evaporation process of the solvent (toluene) after coating. As shown in Figure 6c, combined with the size distribution results in Figure 6d, it can be seen that at experimental temperatures between 20–40 °C, the bubble sizes were mainly small bubbles of 0–100 μm, while above 40 °C, the number of large bubbles above 2000 μm increased significantly, It resulted an obvious increase of bubble area ratio and diameter. This is likely due to the increased solvent evaporation rate caused by the temperature increase. Bubbles do not have enough time to escape the film and coalesce into larger bubbles. Taking into account the bubble density, area ratio, and size distribution, in order to obtain a smooth DCLCEs, the solvent evaporation temperature after coating should be controlled between 20–30 °C, with 20 °C being optimal.

### 2.4. The Modulation of CLCE Film Mechanical Properties via UV Irradiation Dosage During Photopolymerization

For CLCE materials, mechanical properties are key factors determining their application feasibility and service performance. In practical applications, CLCE films often need to withstand various external forces, such as stretching, bending, compression, and torsion. Therefore, CLCE films must possess sufficient strength (Young’s modulus, tensile strength), toughness (elongation at break), and elasticity to resist external forces. In the field of flexible displays, CLCE films need to be able to withstand repeated bending and folding without fracturing. In the field of wearable sensors, CLCE films need to have good tensile properties and resilience to achieve a sensitive response to strain. In the field of smart actuators, CLCE films need to have sufficient strength and driving capability to generate the required deformation or output force. Thus, a systematic investigation into the factors influencing CLCE mechanical properties, coupled with the development of high-performance CLCE materials, is of paramount importance for realizing the widespread application of CLCEs.

The UV radiation dose determines the concentration of free radicals generated by the photoinitiator, thereby controlling the polymerization rate, polymer chain length, and crosslinking density. These microstructural parameters directly determine the mechanical properties of the DCLCEs. A lower UV radiation dose may lead to incomplete polymerization, lower crosslinking density, and thus produce a softer and more fragile film. Conversely, an excessively high dose may lead to over-crosslinking, generate internal stress, and also reduce the toughness of the material, even causing defects. By adjusting the UV radiation dose, the crosslinking network structure of the DCLCEs can be controlled, achieving customized regulation of its mechanical properties. The UV radiation flux (dose rate) and exposure time together determine the total dose, and both need to be precisely controlled to obtain the desired mechanical properties of the DCLCEs.

To systematically investigate the effect of UV radiation flux on the mechanical properties of DCLCEs, we prepared a series of DCLCE samples with different crosslinking densities. The DCLCE precursor solution was uniformly coated onto clean glass substrates using a doctor blade coater (KTQ-II, HENGKAI, Shanghai, China). By controlling the blade gap and coating speed, films with a thickness of approximately 200 μm were fabricated. After complete solvent evaporation, the DCLCEs were photocured using a UV light source (ZF-1, XIUILAB, Shanghai, China) with a UV wavelength of 365 nm and a radiation flux of 30 mW/cm^2^. At each radiation flux, exposure times of 180, 210, 240, 270, and 300 s were set, respectively. The samples were subjected to tensile testing, and the maximum elongation at break was measured.

**Figure 7:**
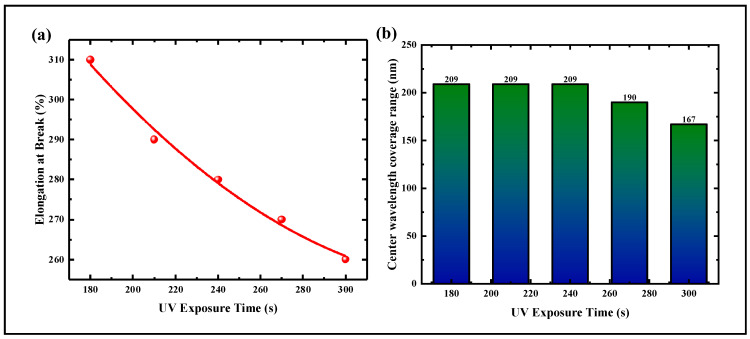
Characterization of mechanical and optical properties under different UV exposure time conditions. (**a**) Elongation at break of DCLCEs under different UV exposure time conditions. (**b**) Coverage range of reflection center wavelength of DCLCEs under different UV exposure time conditions.

Experimental results show that within 180 s, DCLCEs cannot receive sufficient UV radiation to complete photopolymerization thoroughly. When the DCLCEs is stretched, the incompletely self-organized cholesteric phase is disrupted, thus failing to achieve reversible structural color changes. As shown in Figure 7a, when the UV radiation time exceeds 180 s, DCLCEs is completely photocured, but as the irradiation time increases, the DCLCEs becomes brittle and hard, and its ultimate elongation at break decreases, resulting in a smaller coverage range of reflection wavelength.

Experimental results show that when the initial color of the reflective structural color is red, the shift range of the reflection spectrum center wavelength is not affected when the exposure time is between 180 s and 240 s (Figure 7b). When the exposure time is higher than 240 s, the coverage range of the reflection center wavelength decreases. To ensure the maximum spectral coverage range, following the above steps, exposure times of 180, 200, 220, and 240 s were set at each radiation flux, respectively, and the magnitude of stress required under different stretching ratios was measured.

**Figure 8:**
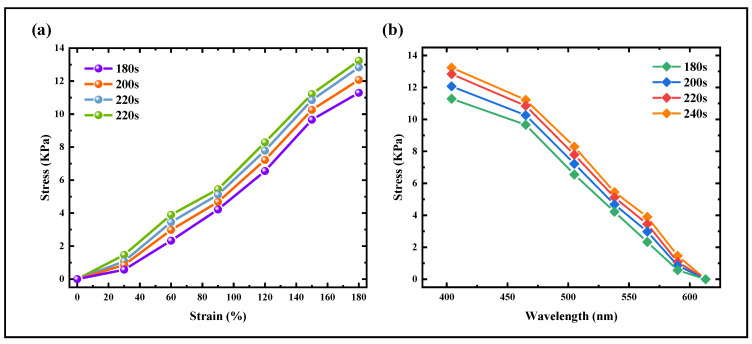
Characterization of mechanical and optical properties while ensuring the maximum spectral coverage range. (**a**) Stress-strain curves of DCLCEs under different UV exposure times. (**b**) Stress-central wavelength changes curves of DCLCEs under different UV exposure times.

As shown in Figure 8, it can be seen that, within the UV exposure time of 180 s–220 s, as the structural color reflected by DCLCEs changes with stretching, the spectral center wavelength coverage range is the same, but the Young’s modulus increases with increasing exposure time. Under the condition of ensuring the maximum spectral coverage range, different UV exposure time fields can be applied to scenarios with different sensitivity requirements. Similarly, by using different masks to control the UV exposure time, patterned and programmable color change can be achieved.

### 2.5. Blade Coating Parameter Optimization: Ensuring Film Uniformity

The thickness uniformity of DCLCEs is crucial for their optical and mechanical properties. To obtain high-quality DCLCEs, we used the blade coating method, and the blade coating process parameters were systematically optimized. Blade coating is a film-forming technique that uses a blade to evenly coat a solution onto a substrate. Its main process parameters include blade height, coating speed, etc. Blade coating parameters directly affect the thickness uniformity and surface smoothness of the film, which in turn affects the uniformity of the structural color. It is necessary to optimize the blade coating parameters to obtain high-quality films.

**Figure 9:**
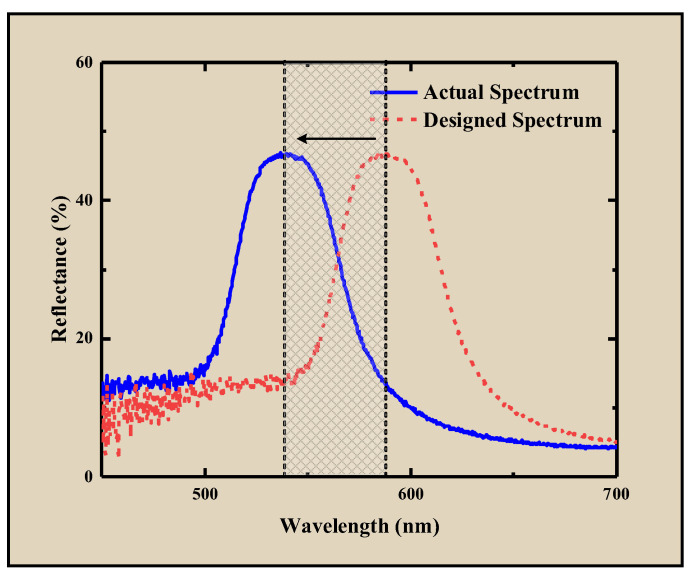
The central wavelength of the reflection spectrum is blueshifted compared to the designed initial structural color. The shaded region indicates the deviation range between the designed and experimentally measured central wavelengths of the reflection spectra. The arrow indicates the shift from the designed spectral central wavelength to the actual spectral central wavelength.

The blade gap is the main parameter that determines the film thickness. If the gap is excessively large, the resulting film is too thick, potentially leading to precursor backflow after coating, creating textures and negatively impacting the structural color. Conversely, an excessively small gap may result in uneven coating or even damage to the substrate. Precise control of the blade gap is therefore essential. The blade height directly determines the initial thickness of the DCLCEs. The lower the blade height, the thinner the DCLCEs; conversely, the higher the blade height, the thicker the DCLCEs. In practice, a precision micrometer screw gauge is used to adjust the distance between the blade and the substrate to precisely control the blade height, ensuring that the wet film thickness meets the requirements. Through preliminary experiments, we determined 100–300 μm as the pre-selected range, and further optimization was performed within this range. Experiments found that when the coating thickness is less than 150 μm, the central wavelength of the reflection spectrum is significantly blueshifted compared to the designed initial structural color, as shown in Figure 9. Five different positions were randomly selected on the prepared DCLCEs to measure the thickness, and the average value and standard deviation were calculated. The smaller the standard deviation, the better the film thickness uniformity.

**Figure 10:**
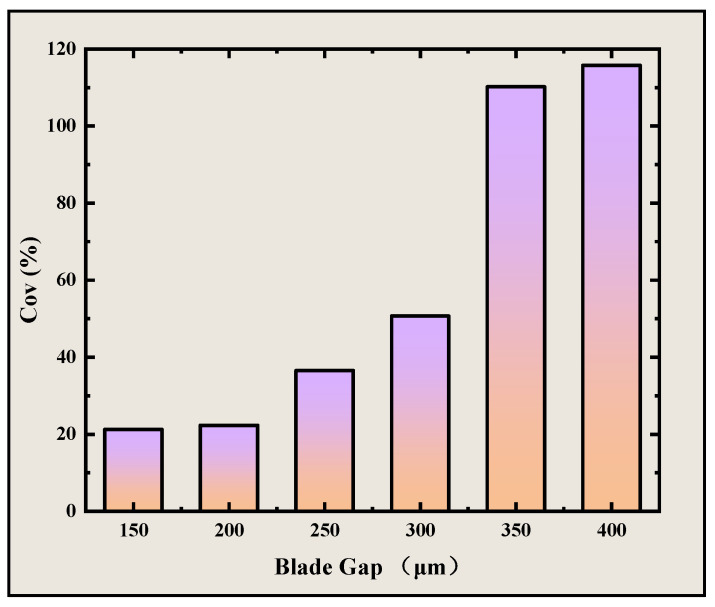
Standard deviation of DCLCEs thickness prepared under different blade gaps.

Experimental results show that when the blade gap is higher than 250 μm, the standard deviation of DCLCEs increases significantly. At blade gaps exceeding 350 μm, the standard deviation surpasses 100%, indicating poor surface flatness (Figure 10). Therefore, the optimal blade gap for coating is determined to be within the range of 150–200 μm.

**Figure 11:**
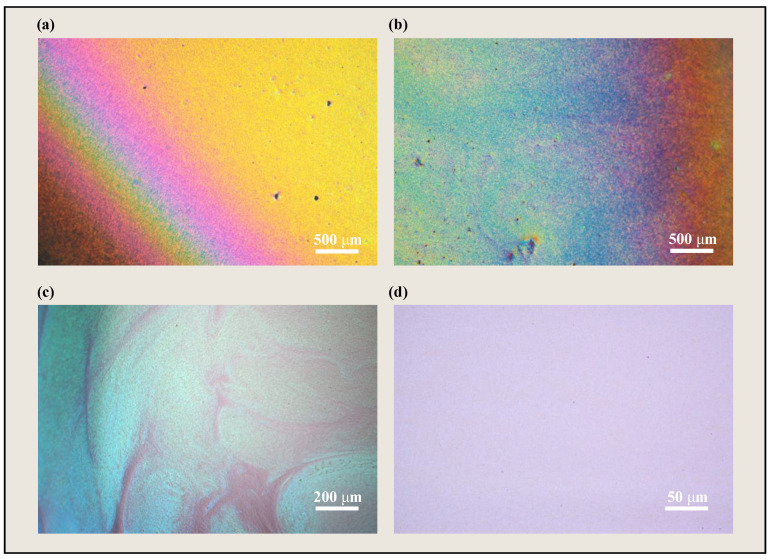
Surface textures of DCLCEs under different coating speeds. (**a**,**b**) Surface textures of DCLCEs under non-uniform speed. (**c**) Surface texture of DCLCEs at 30 mm/s. (**d**) Surface texture of DCLCEs at 20 mm/s.

The coating speed influences both film uniformity and surface quality. When the coating speed is non-uniform, the internal fluid flow can readily generate rainbow-colored band patterns on the sample surface, as illustrated in Figure 11a,b. Under uniform speed conditions, an excessively high coating speed can lead to hydrodynamic instabilities, resulting in defects such as streaks and bubbles (Figure 11c). Conversely, an excessively slow coating speed can cause premature solvent evaporation, hindering the self-assembly process. Optimization of the coating speed is necessary to allow sufficient time for the solution to spread uniformly during the coating process and to form a stable liquid film. The coating speed influences the rheological behavior of the solution and the solvent evaporation rate, which in turn influence the thickness and uniformity of the resulting film. Excessively high coating speeds can lead to film defects such as streaks and bubbles, while excessively low coating speeds can result in excessive solvent evaporation and non-uniform film thickness. Comparative experiments were conducted by controlling the coating speed of the blade coater (KTQ-II, HENGKAI, Shanghai, China), setting it to 10 mm/s, 15 mm/s, 20 mm/s, 25 mm/s, and 30 mm/s, respectively. Pattern analysis, utilizing a gray-level co-occurrence matrix (GLCM), was performed on the resulting samples to determine the roughness of the textural lines.

**Figure 12:**
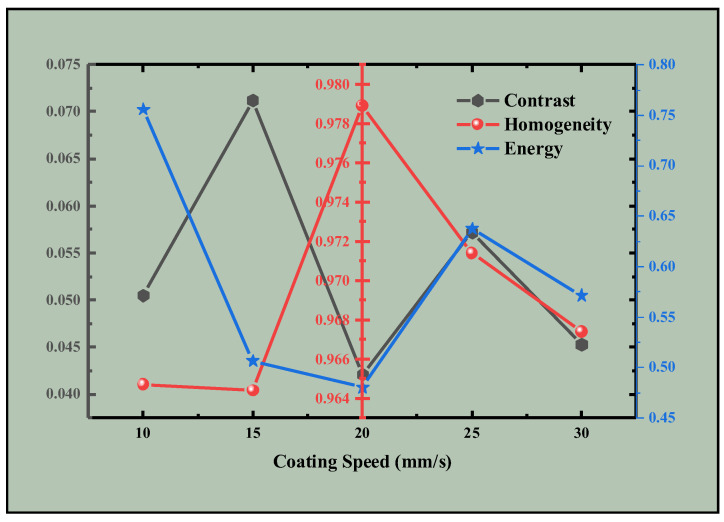
Contrast, homogeneity and energy curves of samples prepared at different coating speeds.

As shown in the data (Figure 12), higher energy corresponds to coarser image texture lines. Greater homogeneity and correlation indicate smoother image regions, with higher correlation signifying greater overall image uniformity. Increased contrast results in a stronger visual impact, with more pronounced and deeper image features. Analysis reveals that the data characteristics align with the visual features of the images. Thus, we conclude that a coating speed of 20 mm/s is the most suitable condition for experimental fabrication.

Through the systematic optimization of the above parameters, we determined the optimal combination of blade coating process parameters: a blade height of 200 μm and a coating speed of 20 mm/s. Under these conditions, the prepared CLCE film has good thickness uniformity.

### 2.6. Characterization of the Optimized Double-Layer CLCEs Structure Performance

**Figure 13:**
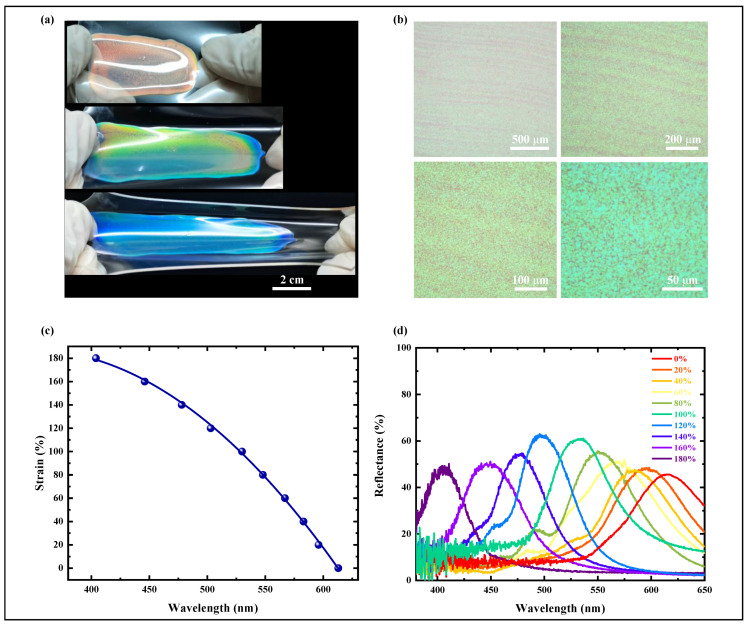
Mechanical and optical properties of DCLCEs. (**a**) Selective reflection color change of DCLCEs under mechanical strain. (**b**) Unstretched DCLCEs observed under an optical microscope at 50×, 100×, 200×, and 500×. (**c**) Fitted curves of the selective reflection center wavelength of DCLCEs under different mechanical strains. (**d**) Blueshift of the selective reflection spectral curves of DCLCEs under different mechanical strains.

To validate the optomechanical performance of the DCLCEs, we observed that the reflected structural color transitioned from red to green, and subsequently to blue, upon the application of increasing tensile mechanical force (Figure 13a,c,d). Measurement of the selective reflection spectra in the DCLCEs was performed using a system comprising a halogen light source (HL2000, Ideaoptics, Shanghai, China), an optical fiber, a high-resolution spectrometer (PG4000, Ideaoptics, Shanghai, China), and a custom-designed fixture. The blueshift of the selective reflection center wavelength of the stretched DCLCEs can be described by the strain within the soft material. Stretching the DCLCEs from its initial length to 2.8 times its original length results in a shift of the center wavelength from 613 nm to 404 nm, providing a tunable bandwidth of up to 209 nm in the visible light range.

The DCLCEs of the samples was examined under an optical microscope (ECLIPSE LV100D, Nikon Corporation, Tokyo, Japan) at magnifications of 50×, 100×, 200×, and 500×. As shown in Figure 13b, the DCLCEs showed controllable color and uniform thickness, with no obvious bubbles or rainbow color changes observed under the microscope. At 200× magnification, the DCLCEs exhibited good monochromaticity, with no obvious streaks or unexpected stray colors.

## 3. Conclusions

This study proposes an improved, simple, efficient, and controllable solvent evaporation-induced self-assembly (SEISA) method for preparing double-layer cholesteric liquid crystal elastomer (DCLCEs) with broadband wavelength tunability, excellent flexibility, and good mechanical properties. We have demonstrated that precise control over the concentration of the chiral dopant LC756 enables precise tuning of the initial pitch of the CLCs’ helical structure, thereby achieving controllable adjustment of the initial reflection color across a broad range (from red to blue). An increase in the LC756 concentration results in a systematic reduction of the helical pitch and a corresponding blueshift of the reflection wavelength. The formation of bubble defects, which significantly reduce the optical performance of DCLCEs, was investigated in detail. Optical microscopy and reflection spectroscopy confirmed that the presence of bubbles reduces reflectivity and disrupts the uniformity of the CLC helical structure. By systematically varying the coating temperature, its crucial role in bubble formation was elucidated. Specifically, a coating temperature range of 20–30 °C (with 20 °C being identified as the optimal temperature) was found to significantly reduce bubble density and area ratio, thereby improving optical quality. The mechanical properties of DCLCEs were systematically tuned by controlling the UV radiation dose during photopolymerization. Within the UV exposure time range of 180 s–220 s, the optimal shift of the reflection spectrum center wavelength can be obtained. As the exposure time increases, the Young’s modulus increases. These microstructural parameters in turn directly affect the macroscopic mechanical properties of DCLCEs. The optimal curing conditions were determined to achieve a balance between sufficient crosslinking for mechanical stability and preventing over-crosslinking, which can lead to brittleness and reduced elongation at break. For coating process parameters, especially blade gap and coating speed, systematic optimization was carried out to achieve excellent film thickness uniformity. A blade gap of 200 μm and a coating speed of 20 mm/s were determined to be the optimal combination, resulting in DCLCEs with minimal thickness variation and enhanced optical uniformity. The optimized double-layer structure of the flexible substrate and DCLCEs allows for a large-scale adjustment of the structural color. Stretching the DCLCEs from its initial length to 2.8 times of its original length results in a shift of the center wavelength from 613 nm to 404 nm, providing a tunable bandwidth of up to 209 nm in the visible light range. The resultant DCLCEs possesses a broad spectrum of adjustable reflective colors, superior flexibility, and robust mechanical properties.

## 4. Optimized Preparation Steps

### 4.1. Preparation of DCLCEs Precursors

First, 4.1 wt% of the chiral reactive mesogenic dopant, 2,5-Bis-O-[4-[[4-[[[4-(acryloyloxy)butoxy]carbonyl]oxy]benzoyl]oxy]benzoyl]-1,4:3,6-dianhydro-D-glucitol (LC756), of the chiral premix was pre-mixed in the achiral diacrylate reactive mesogen 1,4-Bis-[4-(3-acryloyloxypropyloxy)benzoyloxy]-2-methylbenzene (RM257). The prepared chiral premix was dissolved in toluene at a concentration of 50 wt.% at 80 °C for 10 min. Next, a diluted catalyst solution consisting of dipropylamine (DPA) and toluene at a weight ratio of 1:50 was added to the solution and stirred vigorously for 5 min to obtain the final CLCE precursor. The precursor solution was placed in a vacuum chamber at 508 mmHg for 10 min to remove bubbles generated during mixing.

### 4.2. Optimized Coating Process

A clean, elastic substrate (3M™ VHB™ 4910) was placed on a heating platform (WY-01B, Labshark). The heating platform was set to 20 °C and preheated for 30 min to allow the substrate and the heating platform to reach thermal equilibrium. Coating was performed using a coater with a blade gap of 200 μm and a coating speed of 20 mm/s. After coating, the sample was allowed to stand for 24 h under the same temperature and light-protected experimental conditions to completely evaporate the solvent.

### 4.3. Photopolymerization Curing

Following complete solvent evaporation, the CLCE film was subjected to photocuring using a UV light source (ZF-1, XIUILAB), employing a wavelength of 365 nm and a radiant flux of 30 mW/cm^2^ for 180 s, thereby finalizing the DCLCEs preparation.

## Figures and Tables

**Table 1:** The amounts of each precursor component used for each batch of CLCEs.

Chemical Reagent	LC756	RM257	Irgacure 651	Toluene	EDDET	PETMP	DPA
Weight (g)	0.2575	5.1175	0.0355	6.4405	1.6525	0.0610	0.0205

## Data Availability

The original contributions presented in the study are included in the article, further inquiries can be directed to the corresponding author.
